# Overview of anti-viral effects of probiotics via immune cells in pre-, mid- and post-SARS-CoV2 era

**DOI:** 10.3389/fimmu.2023.1280680

**Published:** 2023-12-05

**Authors:** Osamu Kanauchi, Zhao Xuan Low, Kenta Jounai, Ryohei Tsuji, Sazaly AbuBakar

**Affiliations:** ^1^ Tropical Infectious Disease Research and Education Centre (TIDREC), Institute for Advanced Studies, University of Malaya, Kuala Lumpur, Malaysia; ^2^ Institute of Health Sciences, Kirin Holdings Co., Ltd., Fujisawa, Japan

**Keywords:** probiotics, LC-plasma, COVID-19, innate immunity, infectious disease

## Abstract

The COVID-19 outbreak has caused significant global changes and increased public awareness of SARS-CoV-2. Substantial progress in developing vaccines, enhancing sanitation practices, and implementing various measures to combat the virus, including the utilization of probiotics has been made. This comprehensive review examined the medical impact of clinically proven probiotics on infectious diseases, considering three crucial time periods: before (pre-), during (mid-), and after (post-) COVID-19 pandemic era. This review also showed a perspective on the use of probiotics to stimulate the innate immune system and prevent infectious diseases. In pre-COVID-19 era, several probiotic strains were found to be clinically effective in addressing gastrointestinal infectious diseases, the common cold and flu. However, the mechanism by which probiotics exerted their antiviral effects remained relatively unclear during that period. Nevertheless, probiotics, *Lactococcus lactis* strain Plasma (LC-Plasma), and others have gained attention for their unique ability to modulate the immune system and demonstrate antiviral properties. While some probiotics have shown promise in alleviating gastrointestinal symptoms linked to COVID-19, their direct effectiveness in treating or preventing COVID-19 progression has not yet been conclusively established. As we transition into the post-COVID-19 era, the relationship between COVID-19 and plasmacytoid dendritic cells (pDCs), a vital component of the innate immune system, has been gradually elucidated. These findings are now being applied in developing novel vaccines and treatments involving interferons and in immune activation research using probiotics as adjuvants, comparable to CpG-DNA through TLR9. The role of the local innate immune system, including pDCs, as the first line of defense against viral infections has gained increasing interest. Moving forward, insight of the immune system and the crosstalk between probiotics and the innate immune system is expected to highlight the role of probiotics in adjunctive immunoregulatory therapy. In combination with drug treatments, probiotics may play a more substantial role in enhancing immune responses. The immunoregulatory approach using probiotics such as LC-Plasma, which can induce anti-infectious factors such as interferons, holds promise as a viable therapeutic and prophylactic option against viral infectious diseases due to their good safety profile and protective efficacy.

## 1 Introduction

Since the COVID-19 outbreak caused by the severe acute respiratory syndrome coronavirus 2 (SARS-CoV-2) virus, numerous other pathogenic viruses have emerged, and it appears to be an ongoing challenge, with only a few exceptional cases like smallpox (1). As of July 2023, the WHO Coronavirus Dashboard (https://covid19.who.int/) reports that approximately 767 million individuals have been affected by COVID-19, and approximately 7 million lives have been lost to the disease. The global health crisis, emergence of SARS-CoV-2 and its multiple variants, characterized by vaccine resistance, high transmissibility, hypermutation, variable disease severity, and the potential for reinfection has triggered. This situation has led to widespread suffering and loss of life worldwide (2).

It is widely recognized that viruses in general target multiple tissues and organs, leading to a diverse array of symptoms. For example, influenza virus affects the upper respiratory tract and lungs, rotavirus and hepatitis virus affect the gastrointestinal tract (hepatocytes), and HIV primarily infects leukocyte (3). Due to the high rate of virus mutation and the time-consuming process of vaccine delivery, the efficacy of vaccination against some of these viruses infection has been limited. An approach that prioritize activating the innate immune system, which can subsequently activate the acquired immune system, has been proposed. This approach efficiently prevents viral infections, as the activation of innate immunity serves as the first line of defense against viral infections, regardless of the viral species involved (4). In the context of innate immunity, plasmacytoid dendritic cells (pDCs) are one of important candidate as professional antigen-presenting cells within the immune system and play a key role in linking innate and adaptive immunity (5). Furthermore, pDCs are well-known for their ability to upregulate the production of type-1 interferons (IFNs), which act as a first-line defense against infections and prime both innate and adaptive immune responses (6). Studies have reported that type-I IFNs can inhibit viral replication through the induction of interferon stimulated genes (ISGs) from both immune cells and non-immune cells (such as fibroblasts), promote the differentiation and maturation of mDCs, regulate the activation of CD8+ T cells, activate Th1 and NK cells, and induce the primary antibody response field (7). However, the type-I IFN induction was mainly attributed to pathogenic bacteria (8, 9). As a result, the potential of nonpathogenic microbiota, including probiotics, to induce pDC-mediated IFN production for the initial defense against viral infections remains less explored.

Probiotics are defined as live microorganisms that have health benefits for the host (10). They contain various immune-stimulatory stimulants, including lipoteichoic acid, peptidoglycan, and nucleic acid, which act as Toll-like receptor (TLR) ligands, and muramyl dipeptide, which functions as Nod-like receptor ligand (11). Due to their immunostimulatory properties, probiotics have been shown to act on both the innate and acquired immune systems leading to a reduction in the severity of gastrointestinal (12) and upper respiratory tracts (13, 14) infections. Recently, a study revealed that *Lactococcus lactis* strain Plasma (LC-Plasma) could activate human pDCs directly and demonstrated its potential in reducing viral infection symptoms during a clinical trial (15). Thus, probiotic was introduced as a missing link connecting viral infection, the innate immune system, and the acquired immune system.

The COVID-19 pandemic has dramatically changed the world, and there has been a significant shift in people’s awareness of this unknown virus. Alongside the development of vaccines and improvements in the sanitary environment, considerable damage has been incurred, including economic activity restrictions and loss of lives. As we have entered the post-COVID era, efforts are being made to overcome these major challenges. However, the damage caused by COVID-19 is extensive, and in recent years, symptoms of Long-COVID have been observed. Studying the antiviral effects of probiotics before the COVID-19 pandemic is important as these investigations provide a historical foundation for understanding the clinical mechanism of how probiotics can enhance the immune response to a wide range of viral infections in Post-SARS-CoV2 era. Besides, this knowledge is vital for understanding the recently proposed mechanism that links probiotics and innate and acquired immune systems. Therefore, this review aims to examine the clinical effects of clinically proven probiotics on infectious diseases in three stages: Pre-, Mid-, and Post-COVID-19, and provide prospective opinion on future research on infectious diseases using probiotics.

## 2 Probiotic research against infectious disease in pre-COVID-19 era

Compared to the period before the emergence of SARS-CoV-2, the COVID-19 pandemic has resulted in a significantly higher number of victims in a relatively short period of time. During that period, probiotic clinical research primarily focused on common infectious diseases, such as influenza, norovirus, rotavirus, and few others (16). However, among all the probiotic clinical trials, the major focus was on healthy volunteers to prevent infectious viral diseases. Based on our previous review (16), we have provided an updated and concise summary of the results from clinical trials using widely commercial available probiotics, considering both healthy volunteers and patients. For future clinical usage, the effects of probiotics on healthy people and patients should be clearly distinguished. In this section, we very briefly reviewed the efficacy of the commercially available probiotics on viral infection. We present here 11 probiotic bacterial with claimed of clinical utility (**Table 1**), focusing on a recently discovered probiotic, LC-Plasma, which demonstrates anti-viral effects via activating the innate immune system and pDCs. Furthermore, we discussed LC-Plasma clinical efficacy in the context of viral infections.

**Table 1:** Clinical efficacy of commercially available probiotics for viral infectious disease.

Strain	Target disease (symptoms)	Clinical benefit^*^		References
In Healthy Volunteers				
*L.casei Shirota* (LcS)	Epstein–Barr virus (EBV)	Effective		(17)
	Cytomegalovirus (CMV)	Effective		
	Norovirus	NS		(18)
	Upper respiratory tract infection	NS		(19)
*L. delbrueckii ssp. Bulgaricus* (OLL1073R-1)	Common cold symptoms	Effective		(20)
*L. casei* spp. *Paracasei* 431 (L.casei 431)	Response to influenza vaccine	NS		(21)
	Duration of URID symptoms	Effective	
	Influenza incidence/severity	NS		(22)
	Specific IgGs	Effective	
*L. casei* (MCC1849)	Antibody response against vaccination	NS		(23)
*L. casei* (DN-114)	Incidence of acute diarrhea	Effective		(24)
	Incidence of common infectious diseases	Effective		(25)
*L. plantarum* L-137 (HK L-137)	IFN-β, Improvement of QOL	Effective		(26)
	IFN-γ, IL-4, ConA induced prolifereation	Effective		(27)
	Incidence of URID, IFN production	Partial		(28)
*Bifidobacterium animalis Bb12* (Bb12)	Anti-poliovirus specific IgA and anti-rotavirus specific IgA production	Partial		(29)
*Lactococcus lactis* strain Plasma (LC-Plasma)	pDCs acitivity, symptoms of common cold	Effective		(30)
	Anti-viral immune response and physical condition	Effective		(31)
	Incidence rate of influenza	Effective		(32)
	Anti-viral immune response to influenza virus	Effective		(33)
	Dengue-like symptoms	Effective		(34)
	Cumulative school absent days by infectious disease	Effective		(35)
In Patients				
*L. rhamnosus* GG (LGG)	Incident rate of (Experimental) Rhinovirus infection	Partial		(36)
	Acute gastroenteritis (Rotavirus, Cryptosporidium)	Effective		(37)
*Enterococcus faecalis* FK-23	Hepatitis C virus (viral load, ALT)	Effective		(38)
*Saccharomyces boulardii*	Acute rotavirus diarrhea Children	Effective		(39)
*Bifidobacterium lactis* B94	Acute rotavirus diarrhea Children	Effective		(40)

*Effective; statistically significant clinical profit was observed between probiotic and control, NS; not significant difference between groups, Partial; Among parameters, at least one parameter showed significant difference between groups or showed tendency of difference (not significant).

### 2.1 Lactobacillus casei Shirota

LcS was reported to significantly reduce the plasma cytomegalovirus and Epstein-Barr virus antibody titers in highly physically active people. The mechanism of LcS was speculated to be associated with natural killer cells activity which in turn activate cytotoxic lymphocytes and increase T helper 1 cytokine production. This could potentially destruct virus-infected cells and promote antiviral antibody production, thus reducing virus replication (17). While no significant difference in the incidence of norovirus-induced gastroenteritis was observed among long-stay elderly individuals residing at a health service facility, it is noteworthy that an improvement in the intestinal environment occurred. This improvement was characterized by an increase in *Bifidobacterium* and a concurrent rise in the population of *Lactobacillus*, along with an elevation in acetic acid concentration and a decrease in pH.

Although the reason why LcS did not show anti-viral effects was not sufficiently discussed in the report, they speculated it was related to different action of LcS and its metabolites short chain fatty acids to pathogenic bacteria and viruses (18). Unfortunately, LcS had no significant effects on the probability of respiratory symptoms and the influenza-vaccination immune response in healthy elderly nursing home residents. Two potential explanations were considered: first, it was suggested that the advanced age of the individuals in the study might have made them less responsive to the probiotic’s effects. Second, the trial year coincided with an exceptionally mild influenza season, which could have influenced the outcomes (19).

### 2.2 *Lactobacillus delbrueckii ssp. bulgaricus* OLL1073R-1

A study demonstrated that consuming R-1fermented yoghurt augmented NK cell activity and reduced the risk of contracting the common cold in elderly individuals. R-1 and its polysaccharides were believed to increase natural killer cell activity, thus preventing viral infection in the elder person (20).

### 2.3 Lactobacillus paracasei ssp. paracasei

In healthy adults, *L. casei* 431 was found to decrease the duration of upper respiratory symptoms but did not exhibit any impact on the immune response to influenza vaccination. The authors of the study considered the possibility that the immune systems of the elderly participants in their research might not have been responsive to stimulation (21). However, in healthy subjects, *L. casei* 431 was shown to modulate the immune system during vaccination. The probiotic groups exhibited significantly greater increases from baseline in the titers of vaccine-specific IgG, IgG1, and IgG3 in plasma and vaccine-specific secretory IgA in saliva compared to the control group. The mechanism of enhanced Ig production was believed to be a result of microbial changes that led to an altered concentration of signaling molecules, such as short-chain fatty acids (SCFA) or peptides, within the gut lumen. These changes could directly affect the activity of the host’s immune cells. Additionally, it was suggested that direct contact between the host’s immune cells and the gut bacteria may occur, resulting in enhanced Ig production (22).

### 2.4 *Lactobacillus paracasei* MCC1849

In older (≥65) 42 participants in Belgium, the administration of sterilized MCC1849 improved antibody responses to the A/H1N1 and B antigens but did not enhance antibody responses against the vaccination. Small sample size might be the reason why the results could not reach significant differences in this study (23). In another review, it was reported that MCC1849 induces high levels of IL-12 in antigen-presenting cells. This IL-12 acts on naïve T cells, promoting their differentiation into Th1 cells and together with natural killer cells and macrophages, they play a crucial role in innate immunity and are responsible for removing pathogenic bacteria and viruses (41).

### 2.5 *Lactobacillus casei* strain DN-114 001

Supplementation with DN-114 significantly reduced the incidence and frequency of diarrhea compared to the control group when administered to children aged 6–24 months (24). In addition, DN-114 could reduce the incidence of common infectious diseases, including diarrhea, in children aged 3–6 years who attended day-care or school (25). Although the number of fecal *Lactobacillus* positive children in DN-114 was higher than in control, and the decrease in shedding rota virus children was observed, neither of these outcomes reached statistical significance.

### 2.6 *Lactobacillus plantarum* L-137

Sterilized HK L-137 has been found to significantly increased the production of IFN-β in healthy volunteers (26). Hirose et al. assessed the effects of HK L-137 intake over 12 weeks on the quality of life (QOL) and immune functions, such as NK cell activity, IFN-γ and IL-4 production, and concanavalin A (ConA) induced proliferation in healthy volunteers. The results revealed that QOL significantly improved in the HK L-137 group after 8 weeks of intervention, along with a significant increase in IFN-γ production and ConA-induced proliferation while reducing the production of IL-4 (27). In another trial, HK-137 significantly reduced the incident rate of upper respiratory infectious diseases (URID) in healthy volunteers, though it did not lead to changes in IFN-β production and IL-4 levels (28).

### 2.7 *Bifidobacterium animalis* Bb12

When infant starter formula containing Bb12 was administered to six-week-old healthy participants for 6 weeks, Bb12 was reported to improve intestinal immunity. The study showed a significant increase in anti-poliovirus-specific IgA production and a mild enhancement in anti-rotavirus-specific IgA production (P =0.056). Bb12 demonstrates the potential to alleviate negative immune-related effects resulting from the absence of breastfeeding and cesarean-section delivery. It offers infants a safe and dietary means of experiencing immune-modulating bacteria (29).

### 2.8 Lactobacillus rhamnosus

In a study where subjects were intranasally inoculated with rhinovirus following after 6 months of LGG oral treatment observed a reduction in the frequency and severity of cold symptoms, although the results were not statistically significant (36). In another study, LGG was administered for 4 weeks to children with gastroenteritis, who tested positive for either rotavirus or Cryptosporidium species in the stool, results demonstrated that LG significantly increased the serum immunoglobulin IgG in children with rotavirus-induced diarrhea after the intervention. On the other hand, in children with cryptosporidial diarrhea, LGG significantly improved intestinal permeability, along with an increase in serum IgG and secretory IgA levels. LGG might restore intestinal integrity in children with diarrhea cause by Cryptosporidium or rotavirus infection (37). Since these studies involved infected participants, we categorized them as studies with patients in **Table 1**.

### 2.9 Enterococcus faecalis

FK-23 significantly reduced alanine aminotransferase (ALT) levels in adult HCV-positive subjects but did not decrease the viral load. Although the detailed mechanism is still not fully understood, based on their previous study, it was suggested that oral administration of FK-23 might have the potency of regulating the balance of the intestinal microflora and decrease serum ALT in HCV patients (38).

### 2.10 Saccharomyces boulardii

Oral administration of *Saccharomyces boulardii* and rehydration significantly shortened the duration of diarrhea in acute rotavirus gastroenteritis children in Bolivia, compared with control rehydration alone (39). Although the detailed mechanism in this study was not elucidated, other study suggested that increase production of short-chain fatty acids in colonocytes, reduction in the permeability of the intestinal barrier, decrease in the invasion of microorganisms, high levels of IgA and IL-10 in the bowel might be the beneficial physiological effects of *Saccharomyces boulardii* which might lead to protection against acute rotavirus gastroenteritis (42).

### 2.11 *Bifidobacterium lactis* B94

B94 with oral rehydration treatment significantly shortened the diarrheal period in acute rotavirus gastroenteritis infants/children (5 months to 5 years old), compared with control oral rehydration alone (40). *In vivo* study reported that B94 improved the gut microbiota and metabolic dysbiosis, thus reducing pathological abnormalities in gastrointestinal organs (43).

### 2.12 *Lactococcus lactis* strain plasma

#### 2.12.1 Anti-viral infection by pDCs and type-I IFNs production

Previous reports indicated that microbiota-induced pDC activation typically resulted from pathogenic microbes, such as the spherical bacterium Staphylococcus aureus. However, edible LC-Plasma was the first probiotic to demonstrate direct activation of pDC. As a result, this review focused on the anti-viral effects of LC-Plasma via activation of pDC. In the response against viral infections, innate immune cells such as DCs and pDCs play crucial role as key “liaisons” bridging the innate and acquired immune responses. They accomplish this by recognizing pathogenic and endogenous inflammatory signals, facilitating communication between the two arms of the immune system (44). During viral infection, pDCs function as professional antigen-presenting cells and utilize TLR7 and TLR9 as crucial components of their endosomal nucleic acid sensing mechanism, thus allowing them to detect the presence of bacterial and viral nucleic acids. Upon recognition, pDCs initiate a signaling cascade that produces type-I and type-III IFNs and other cytokines and chemokine (45).

#### 2.12.2 Direct activation of pDC by LC-Plasma

It was demonstrated that using DCs derived from Flt-3L-stimulated murine bone marrow obtained from TLR knockout mice, LC-Plasma could stimulate the production of type-I IFNs by pDCs and myeloid DCs. These findings suggest that LC-Plasma has the potential to directly activate DCs and induce the production of type-I and type-III IFNs. Subsequent studies revealed that the IFN-α production was completely abolished in pDC obtained from TLR9 or MyD88 knockout mice, suggesting that LC-Plasma directly stimulated IFN-α production via TLR9/MyD88 signaling pathway. Additional evidence supports this observation, as IFN-α production was induced not only by CpG DNA (a TLR9 agonist) but also by DNA extracted from LC-Plasma (7). In addition, LC-Plasma was observed to be specifically internalized by pDC, highlighting that phagocytosis of LC-Plasma by pDCs is crucial to activate pDCs and enhance the production of IFNs (7, 46). Additionally, the production of IFN-α by pDCs was synergistically elevated when co-cultured with mDCs in the presence of LC-Plasma. This suggests that crosstalk or direct contact between mDCs and pDCs is necessary for effective induction of IFN-α production. The interaction between these DC subsets appears to play a crucial role in enhancing the immunostimulatory effects of LC-Plasma and the subsequent IFN response (7).

#### 2.12.3 Anti-viral infectious effects of LC-Plasma from pre-clinical study

In an *in vivo* experiment, oral administration of LC-Plasma before the parainfluenza virus (mPIV1) challenge resulted in a significantly higher survival rate in mouse. This was accompanied by an upregulation of genes with antiviral activities and a reduction in lung inflammation compared to the control group. These findings suggest that LC-Plasma intake may confer protective effects against mPIV1 infection, improving survival and reducing lung inflammatory response (47). In another study, when mice were orally administered with LC-Plasma for 2 weeks before the dengue virus (DENV) challenge, there was a significant decrease in relative viral titers, resulting in a reduction in the expression of inflammatory genes compared to the control group. For example, IL-6, MCP-1, and IFN-γ expression were reduced in the splenocytes, and expression of TNF-α was reduced in the liver cells. These findings suggest that LC-Plasma administration has a positive impact on controlling DENV infection and modulating the inflammatory response in the spleen and liver, leading to potential benefits in reducing the severity of the DENV infection (48).

#### 2.12.4 Clinical trials of LC-Plasma for infectious disease

**Figure 1:**
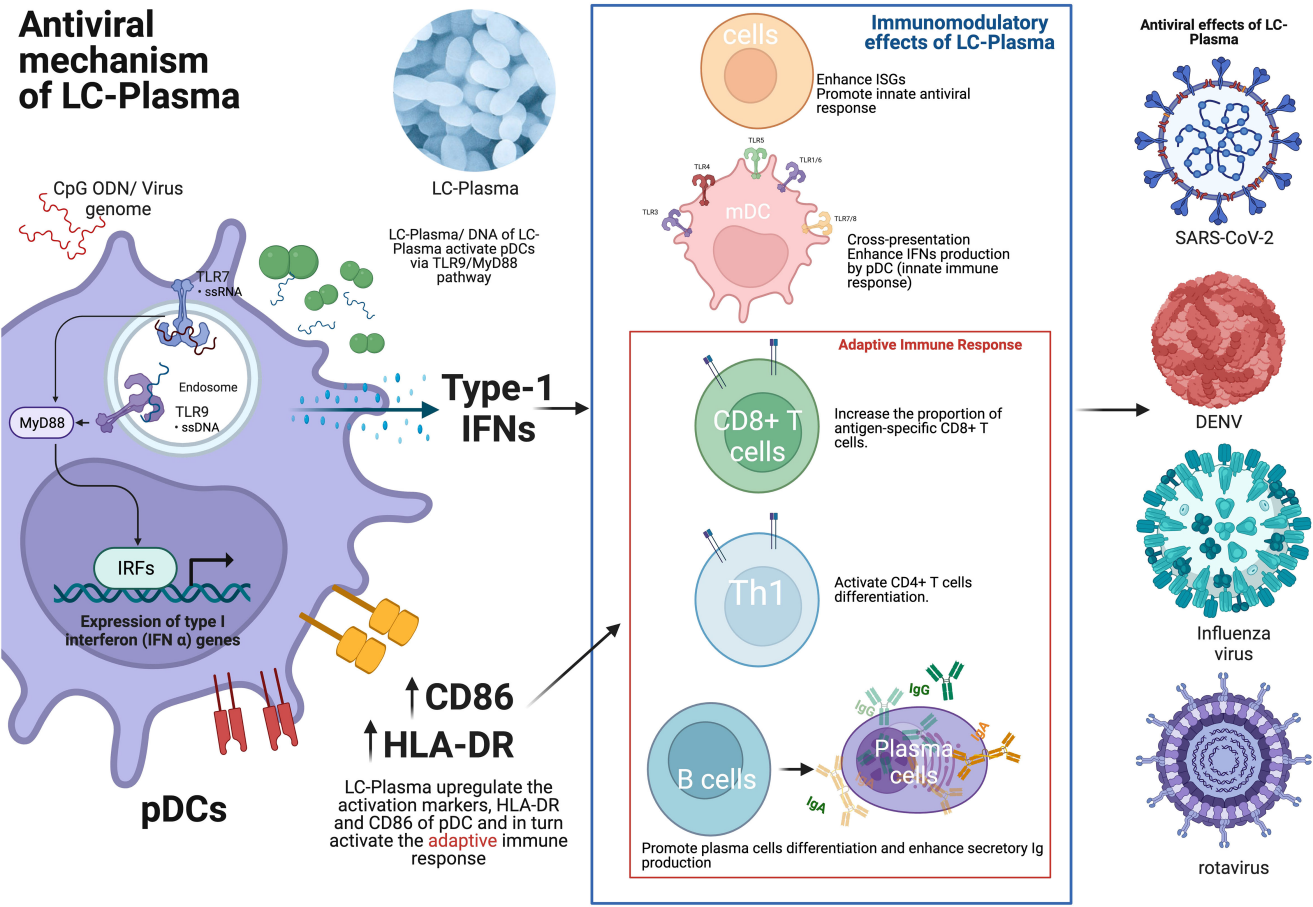
The antiviral mechanism of LC-Plasma. LC-Plasma activates the pDCs via TLR9/MyD88 pathway and upregulates the type-I IFNs production, which subsequently activate the adaptive immune response against viruses. LC-Plasma, *Lactococcus lactis* strain Plasma; TLR, Toll-like receptors; MyD88, Myeloid differentiation primary response 88; IFNs, Interferons; IRFs, Interferon regulatory factors; ISGs, Interferon stimulative genes; pDCs, Plasmacytoid dendritic cells; mDC, Myeloid dendritic cells; Ig, Immunoglobulin; SARS-CoV-2, Severe acute respiratory syndrome coronavirus 2; DENV, Dengue virus. (Picture was Created with BioRender.com).

Oral administration of LC-Plasma activated human pDCs among PBMCs from healthy volunteers, especially in a subgroup of volunteers who originally showed a low pDC activity. LC-Plasma also significantly attenuated cumulative common cold symptoms score (30). It was reported that there were prophylactic effects of LC-Plasma on influenza-like illness in healthy volunteers in a study done during the winter season. Significant decrease in the cumulative number of days of incidence of major symptoms of an influenza-like illness were observed. Increase in IFN-α gene expression of PBMC isolated from healthy volunteers who orally consumed LC-Plasma for 12 weeks were observed (31). In addition, a 4-week oral intake of LC-Plasma also leads to upregulating pDC activation markers, namely HLA-DR and CD86 (30). Sakata et al. reported that LC-Plasma significantly reduced the cumulative incidence rate of influenza among schoolchildren in Japan (32). Fujii et al. reported that oral administration of LC-Plasma demonstrated prophylactic effects in healthy volunteers during the winter season in Japan. Specifically, it increased secretory IgA levels in saliva and maintained the activation level of pDC with CD86 positive (33). Finally, it was reported that LC-Plasma significantly attenuated Dengue fever-like symptoms in Malaysian healthy adult volunteers (34) and significantly reduced the cumulative school absent days by infectious disease in Vietnam elementary school children (35). **Figure 1** shows the overview of the immunomodulatory effect and the antiviral mechanism of LC-Plasma.

### 2.13 Brief summary and point of view in pre-COVID-19 era

In the period pre-COVID-19, the mechanisms through which probiotics exerted their antiviral effects remained unclear. Nevertheless, certain probiotics, such as LC-Plasma, and others have gained attention for their unique physiological properties. In clinical trials involving patients with viral infections, most probiotics demonstrated clinical benefits, with a particular focus on gastroenteritis-related diseases (**Table 1**). In general, clinical trials involving healthy volunteers targeted a wide range of symptoms, including upper respiratory infectious diseases, but some of these trials did not show significant efficacy. Major probiotics are commonly found amongst the intestinal microbiota and fermented foods that are a part of daily diet. This close association allows probiotics to easily interact with immune cells via the intestinal mucosa, including structures like Peyer’s patches and Microfold cells. As a result, probiotics can directly exert their effects at these sites, such as reducing diarrhea and infectious viral replication. In contrast, probiotics may be more challenging to impact other organs such as the upper respiratory tract directly. Recent research has highlighted the Gut-Lung axis concept, which involves cross-talk (bidirection) between the gut and the lung. From gastrointestinal tract to lung, immune cells in the intestinal (sub) mucosa or the mesenteric lymph nodes are reported to pivotal role in translocating microbiota and related fragments/metabolites to travel along the mesenteric lymphatic system to pulmonary circulation. Moreover, intestinal microbiota is critical for maintenance of T cell subsets that are important for systemic immunity (e.g. expansion of CD4+ T cells, regulatory T cells, Th1/Th2 responses, and Th17 T cells) (49). The alteration of the ratio of *Firmicutes* and *Bacteroidetes* directly influences the composition of gut microbiota metabolites, leading to changes in the concentration of circulating short-chain fatty acids. For instance, microbial-produced butyrate has been reported to exert a wide range of anti-inflammatory activities. It affects immune cell migration, adhesion, cytokine expression, as well as cellular proliferation and activation (50). It is also reasonable to speculate that this axis operates in a similar manner when it originates in the lung mucosa and lung lymph nodes. This axis has been reported to demonstrate a not unidirectional but bidirectional pathway, as evidenced by non-absorbable tracer deposited into the nasal cavity being found in the gastrointestinal (GI) tract within 60 minutes in mice (51).

Notably, in conditions such as chronic obstructive pulmonary disease and sepsis, where the immune system is compromised, there is evidence suggesting that dysbiosis and increased gut permeability may result in the translocation of gut bacteria or their components to the bloodstream and subsequently to the lungs (52). Considering these fundamental benefits, exploring probiotics as a therapeutic option for mitigating COVID-19 becomes crucial. Modulating the gut microbiota through probiotic administration holds promise in controlling respiratory barrier integrity. Alongside vaccinations, antiviral medications, oral intake of probiotics might be invaluable for ameliorating COVID-19.

## 3 Probiotic research against infectious disease COVID-19 in mid-COVID-19 era

### 3.1 SARS-CoV2

SARS-CoV-2 is a group of enveloped positive single-stranded RNA genome viruses. Its genome encodes 3 structural proteins (S protein, E protein, M protein, and N protein) and 16 non-structural proteins (NSPs). The S protein primarily facilitates the virus’s entry into host cells. SARS-CoV-2 gains entry by using the S1 subunit of the S protein to bind with the host receptor angiotensin-converting enzyme 2 (ACE2), which is expressed on the host cell membrane. This receptor is predominantly found in the epithelial cell membranes of the lungs, mucosa, and small intestines. Person-to-person virus transmission relies on aerosol droplets released from an infected individual (53).

### 3.2 pDC, activation, IFN-α production and COVID-19 pathogenesis

pDCs play a crucial role in innate immunity due to their ability to produce many type-I IFNs. They express high levels of endosomal nucleic acid sensing receptors, specifically TLR7 and TLR9, which enable them to detect nucleic acids from pathogens. In response to these signals, pDCs secrete a substantial amount of IFN-α and various cytokines and chemokines, contributing to the rapid inflammatory response, characteristic of innate immunity (45). Innate immunity is the primary defense against external injury, self-abnormalities, and infections caused by pathogenic microorganisms or viruses. The innate immune system quickly and broadly responds to counteract potential threats. pDCs play a crucial role in this response, as they directly contribute to pathogen clearance and activate adaptive immune responses (44). During acute SARS-CoV-2 infection, there is a notable reduction in pDC levels and a considerable decrease in IFN-α production in infected patients (54). This reduced IFN-α production has been identified as a significant parameter associated with disease severity, supporting its potential use as an early biomarker for disease progression. Understanding the role of pDCs and their IFN-α production in relation to SARS-CoV-2 infection can offer valuable insights for developing targeted therapies and monitoring disease outcomes (55).

### 3.3 Clinical trial of probiotics for COVID-19

At present, it is difficult to conclude the efficacy of probiotics in general based on systematic review or meta-analysis, since numerous large-scale clinical trials on COVID-19 are still ongoing and have not been completed yet due to the serious conditions brought about by the pandemic (56). As the COVID-19 pandemic progresses, the emergence of diverse SARS-CoV-2 variants with distinct characteristics, such as high mutation rates, varying levels of virulence, and transmissibility, along with the implementation of different vaccination strategies (types and timing of vaccination) in various regions, poses significant challenges when analyzing clinical studies related to the virus (57).

Initially, there were available nine clinical trials on probiotics for COVID-19 in recent meta-analysis study (58), but two studies were excluded as they involved the use of antibiotics as part of the standard therapy (59, 60), while two other studies were excluded due to their retrospective design (61, 62). To minimize potential biases and ensure the quality of the discussions, five clinical trials were included in the this section. This selection process allows for a more focused analysis of the relevant clinical trials on probiotics for COVID-19, potentially providing valuable insights into their potential efficacy and role in managing the pandemic.

### 3.4 Florasan^®^ - D (Valenta Pharm, Russia)

The probiotic supplement, Florasan^®^ - D, contains four strains of probiotics: *Lacticaseibacillus rhamnosus* PDV 1705, *Bifdobacterium bifdum* PDV 0903, *Bifdobacterium longum* subsp. *infantis* PDV 1911, and *Bifdobacterium longum* subsp*. longum* PDV 2301 with each strain at approximately ~10^9^ colony forming units (CFU), respectively. In an open-label trial conducted on inpatients with diagnosed COVID-19 and pneumonia, the participants were randomly assigned to the Control group (n=101) and Probiotics group (n=99). They were required to take one capsule three times a day. The study aimed to assess the effects of probiotic supplementation on various clinical outcomes in COVID-19 patients. The results indicated that there were no significant differences between the control and probiotics groups in terms of mortality, total duration of the disease, hospital stay, incidence of intensive care unit admission, need for mechanical ventilation or oxygen support, liver injury development, or changes in inflammatory biomarkers. However, it was observed that the probiotics group had significantly shorter admission days with diarrhea than the control group (63).

### 3.5 Mixture of *Kluyveromyces marxianus* B0399 and *Lactobacillus rhamnosus* CECT 30579

In a prospective, open-label, case-control intervention study, the researchers assessed the effects of oral intake of the probiotics *Kluyveromyces marxianus* B0399 (1 × 10^9^ CFU/capsule) and *Lactobacillus rhamnosus* CECT 30579 (1 × 10^8^ CFU/capsule) on the progression of COVID-19 patients. During the course of the study, all patients were allowed to receive standard COVID-19 treatments. The Probiotic group received the probiotic mixture once a day for a duration of 30 days, in addition to their regular COVID-19 therapy (n=24), while the control group only received the standard COVID-19 therapy without any probiotic supplementation (n=15). Results from the study showed that the Probiotic group exhibited a notable reduction in the number of patients experiencing pyrosis (heartburn), abdominal pain, and musculoskeletal pain compared to the control group. Additionally, there was a significant improvement in the overall number of symptoms (both digestive and non-digestive) in the Probiotic group, suggesting a potential positive impact of probiotic supplementation on symptom management during the course of the study (64).

### 3.6 AB21^®^ (AB-BIOTICS, Spain)

AB21^®^ is a probiotic formula containing a 1:1 ratio of *Lactiplantibacillus plantarum* strains (KABP022, KABP023, and KAPB033) and *Pediococcus acidilactici* KABP021, with a total of 2 × 10^9^ CFU per capsule. In a single-center, quadruple-blinded, randomized trial involving adult symptomatic COVID-19 outpatients, the probiotic group (n=147) was instructed to consume one AB21^®^ capsule per day for a duration of 30 days, while the control group (n=146) received Maltodextrin. The study findings revealed a significant difference in complete remission between the AB21^®^ group (53.1%) and the control group (28.1%). Furthermore, AB21^®^ was observed to significantly reduce nasopharyngeal viral load, lung infiltrates, and the duration of both digestive and non-digestive symptoms compared to the control group. Although no significant changes were observed in the fecal microbiota between the two groups, AB21^®^ significantly increased the levels of specific IgM and IgG antibodies against SARS-CoV-2 compared to the control group. Based on these results, the authors hypothesized that AB21^®^ primarily acts on the host’s immune system via the gut-lung axis, potentially contributing to its beneficial effects in managing COVID-19 symptoms and promoting recovery in symptomatic outpatients (65).

### 3.7 LactoCare^®^ (Zist Takhmir, Iran)

In a randomized placebo-controlled trial, researchers enrolled hospitalized patients with COVID-19 to investigate the effectiveness of the synbiotic supplement LactoCare^®^ on clinical and paraclinical outcomes. LactoCare^®^ contains a combination of beneficial microorganisms, including *Lactobacillus rhamnosus*, *Lactobacillus casei*, *Lactobacillus acidophilus*, *Bifidobacterium breve*, *Lactobacillus bulgaricus*, *Bifidobacterium longum*, and *Streptococcus thermophiles*, with a concentration of ~10^9^ CFU per capsule. During the study, the probiotic group (n=38) received LactoCare^®^ twice daily for a period of 2 weeks, while the control group (n=38) received fructooligosaccharides as a placebo. Although there were no significant differences observed in clinical and paraclinical symptoms between the two groups, it was found that the serum inflammatory cytokine, IL-6, showed a significant decrease in the LactoCare^®^ group compared to the control group after the 2-week intervention (66).

### 3.8 Lactibiane Iki^®^ (PiLeJe Laboratoire, France)

A prospective randomized controlled trial aimed to assess the effectiveness of a probiotic mixture, Lactibiane Iki^®^, in reducing fecal calprotectin (an inflammatory marker) as its primary endpoint, and its secondary endpoint was to evaluate the reduction in oxygen support and length of hospital stay in patients with COVID-19 pneumonia compared to a control group. The probiotic mixture Lactibiane Iki^®^ contains three strains: *Bifidobacterium lactis* LA 304 at 6 × 10^9^ CFU, *Lactobacillus salivarius* LA 302 at 28 × 10^9^ CFU, and *Lactobacillus acidophilus* LA 201 at 6 × 10^9^ CFU per sachet. Patients in the Lactibiane Iki^®^ group (n=40) received the probiotic mixture twice a day for a duration of 10 days, in addition to standard therapy, while the control group (n=40) received standard therapy only. The study found that the fecal calprotectin levels in the Lactibiane Iki^®^ group were significantly lower compared to the control group, indicating a potential reduction in inflammation. However, no significant differences were observed between the two groups regarding oxygen support requirements and length of hospital stay. Unfortunately, clinical symptom data was not available in this study (67).

### 3.9 Brief summary and lesson from probiotics clinical trials in mid-COVID-19 era

The COVID-19 pandemic has had a significant impact on clinical trials undertaking worldwide, leading to disruptions such as difficulties in conducting trials, site closures, travel limitations, and interruptions in the supply chain for investigational products (68). Despite these challenges, several probiotics have been studied for their potential effects on COVID-19 patients. Florasan^®^-D did not show improvement in COVID-19 symptoms and outcomes, but it significantly reduced the incidence of diarrhea in patients. The probiotic mixture containing *Kluyveromyces marxianus* B0399 and *Lactobacillus rhamnosus* CECT 30579 showed promising results in reducing symptoms such as pyrosis, abdominal pain, and musculoskeletal pain, as well as improving overall symptoms. AB21R demonstrated a higher complete remission rate and significant reductions in viral load, lung infiltrates, and digestive and non-digestive symptoms duration. LactoCare^®^ showed a significant decrease in the inflammatory cytokine IL-6 in patients. Additionally, Lactibiane Iki^®^ was found to attenuate the inflammatory marker fecal calprotectin. While the immune mechanisms of these probiotics in combating COVID-19 were not fully elucidated in these 5 clinical studies, they have shown potential in improving gastrointestinal symptoms and displaying anti-inflammatory effects as adjunctive therapeutic options. Despite limitations, large-scale studies on a global scale are necessary to obtain more robust evidence, including understanding the crosstalk between host immune cells and SARS-CoV-2. The International Scientific Association of Probiotics and Prebiotics strongly recommends further investigation into the relationship between gut microbiota and susceptibility to COVID-19 and the role of various probiotic strains in lowering viral load through different mechanisms (56).

### 3.10 LC-Plasma research for COVID-19 in mid-COVID-19 era

In animal studies, LC-Plasma was found to activate pDCs and induce the production of type-I and type-III IFNs, which are essential in fighting parainfluenza virus infections (47). Additionally, when pDCs were stimulated by LC-Plasma *in vitro*, the culture supernatant significantly suppressed the replication of SARS-CoV-2, suggesting potential antiviral effects (69).

In addition, LC-Plasma treatment has demonstrated the ability to enhance various aspects of the immune response. It increases the proportion of antigen-specific CD8+ T cells, which are crucial for targeting and eliminating virus-infected cells, and activates CD4+ T cells, which play a pivotal role in coordinating and regulating the immune response (**Figure 1**). Furthermore, LC-Plasma has been shown to stimulate humoral immune responses, particularly by increasing the production of antigen-specific IgG antibodies. These antibodies are essential for recognizing and neutralizing viruses, thus providing an important defense against infections (70). (**Figure 1**). Therefore, it is hypothesized that LC-Plasma, which can upregulate pDCs, may stimulate humoral immune responses through CD4+ T cell differentiation and simultaneous interactions between T and B cells. Based on these promising findings, a clinical trial of LC-Plasma for COVID-19 patients with asymptomatic or mild symptoms were conducted and is currently still ongoing (71). The aim of this trial is to relieve or prevent COVID-19 symptoms in these patients, leveraging the immune-boosting properties of LC-Plasma observed in earlier preclinical studies.

## 4 Probiotics in the post-COVID-19 era

The COVID-19 era has witnessed the widespread implementation of various public health and social measures, including the use of personal protective measures like mask-wearing, hand hygiene, and social distancing, as well as environmental measures and surveillance and response protocols. These measures have played a crucial role in mitigating the transmission of SARS-CoV-2. However, they have also significantly impacted other respiratory viral diseases. Many studies have reported a remarkable decrease in the incidence of common respiratory pathogens during the COVID-19 pandemic. Interestingly, after lifting of the COVID-19 countermeasures, some countries experienced a shift in seasonality and a delayed outbreak of respiratory syncytial virus (RSV), with more infected patients (72).

Analyzing numerous independent clinical trials into a single meta-analysis is undeniably challenging due to various confounding factors, such as age, dietary conditions, microbiota, genetic and epigenetic immune status of individuals, study season, and variable viral epidemiology. These factors influence the outcomes and conclusions of the studies, and standardizing them is difficult. Nevertheless, some meta-analyses have suggested that probiotics may be beneficial in preventing and treating upper respiratory viral infections (73). The mechanism of action of probiotics in the context of SARS-CoV-2 infection extends beyond conventional meta-analyses aimed at symptom relief. This is because probiotics have multiple roles in influencing the host. Systematic Network and Meta-analysis, in conjunction with MCODE (Molecular Complex Detection) cluster analysis, has revealed the multifunctional effects of probiotics on SARS-CoV-2 infection. This analysis identified eleven MCODE clusters, including functions related to the mucosal barrier, immune activation, innate immune responses, glucose/lipid metabolism, and more, all exhibiting significant changes (74).

### 4.1 Therapeutical/prophylactic option against viral infection in the post-COVID-19 era

In the Post-COVID-19 pandemic, we propose two strategies to manage emerging viral infections effectively. Firstly, there is a need to explore more feasible medical therapeutic options against newly emerging SARS-CoV-2 variants and influenza. This involves accelerating current vaccine research to develop vaccines that can effectively cover new strains and variants. Besides, it is also important to focus on developing broad-spectrum antiviral drugs, improving cytokine therapy, and refining blocking agents for better efficacy. These strategies require a deeper understanding of the critical role of innate immunity in viral infections and the crosstalk between viral components and immune cells. Despite substantial advancements and positive results from vaccine candidate trials, several challenges still exist. These challenges include potential adverse effects of vaccines, the need for an ultra-cold supply chain for mRNA vaccines, and the weak immunogenicity of recombinant antigens even with the presence of aluminum salts as adjuvants (75). Despite these obstacles, vaccines are still being delivered worldwide to prevent virus-related diseases (76). To effectively combat emerging viral infections, however, a comprehensive approach that includes a combination of vaccination, antiviral drugs, and a better understanding of innate-acquired immunity is essential.

### 4.2 Current COVID-19 vaccines

During COVID-19 pandemic, the acceleration of the COVID-19 vaccine development was expedited, and moreover, the mRNA platform was already being used. Although conventional vaccine research and development needs a long process, many COVID-19 vaccines are launched and saved our lives. The current COVID-19 vaccines are based following platforms: protein subunit, DNA, RNA, non-replication viral vector, replicating viral vector, inactivated virus, virus-like particles, live attenuated virus, replicating viral vector combined with an antigen-presenting cell, non-replication viral vector combined with an antigen-presenting cell, and bacterial antigen-spore expression vector. It was reported that protein subunit vaccines and RNA vaccines were major (77).

Among them, Zhou et al. reported that RNA vaccines possessed several advantages, such as high efficacy, adaptability, simplicity in antigen design, and the ability to induce both humoral and cellular immunity and that RNA vaccines could offer relatively rapid and cost-effective manufacturing, flexibility to target emerging or mutant pathogens and a potential approach for clearing immunotolerant microbes by targeting bacterial or parasitic survival mechanisms (78). However, mRNA vaccines had to be formulated in lipid nanoparticles for intramuscular injection, and the ionizable lipids, which are a necessary component of lipid nanoparticles for effective cell transport, have the potential to cause adverse events. (e.g. polyethylene glycol formulations can allergic reactions). To mitigate this issue, it remains necessary to search for more cost-effective adjuvants (79).

### 4.3 Immune boosters as vaccine adjuvants

**Figure 2:**
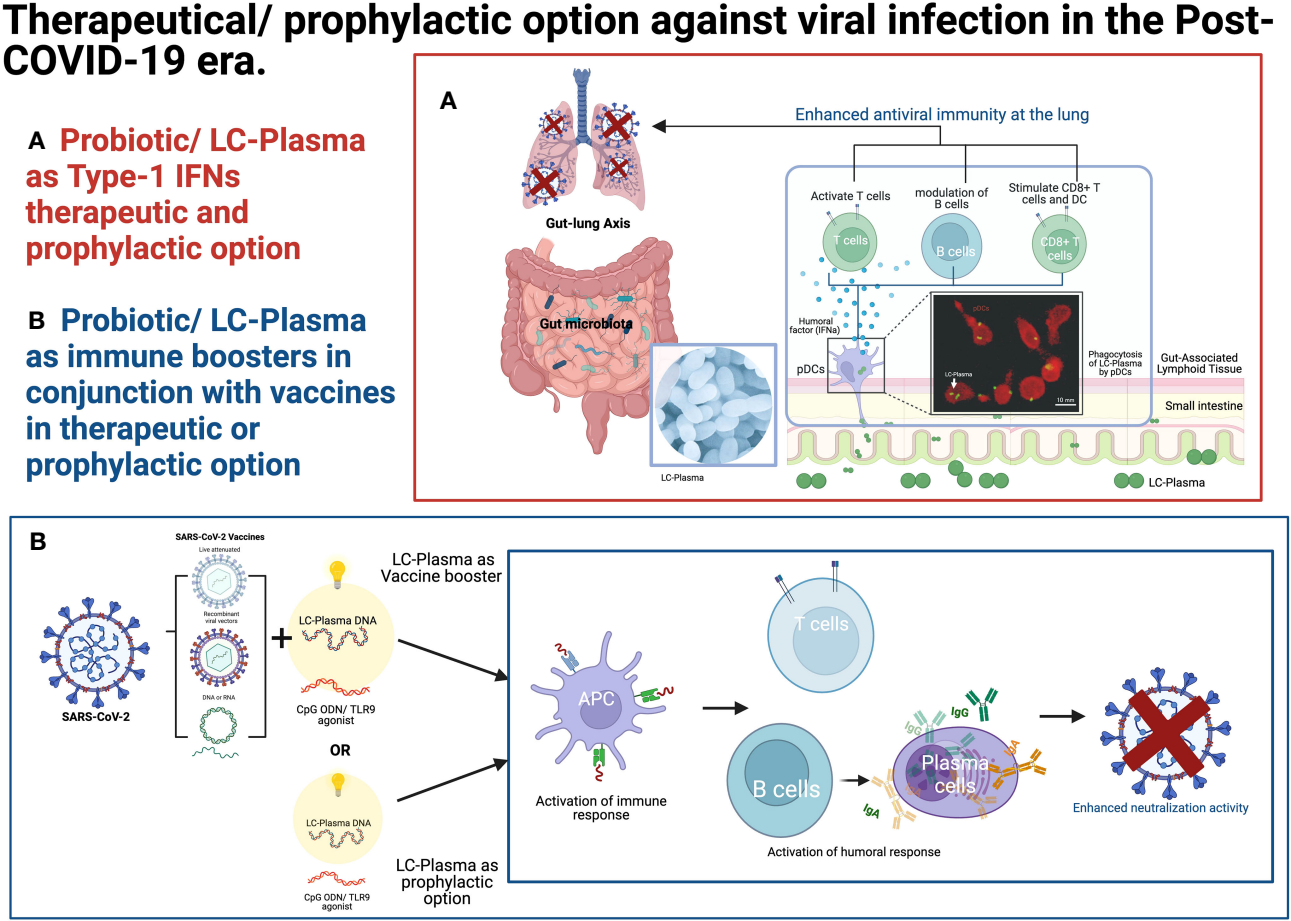
Therapeutical/prophylactic option against viral infection in the post-COVID-19 era. **(A)** Probiotics/LC-Plasma might offer an alternative therapeutic and prophylactic option for regulating the endogenous Type-1 IFN levels. **(B)** Probiotics/LC-Plasma might serve as an alternative therapeutic or prophylactic option for boosting the immune system in conjunction with vaccines. The fluorescence microscopic image was adopted from the previous publication (47). LC-Plasma, *Lactococcus lactis* strain Plasma; IFNs, Interferons; ISGs, Interferon stimulative genes; pDCs, Plasmacytoid dendritic cells; SARS-CoV-2, Severe acute respiratory syndrome coronavirus 2; DNA, deoxyribonucleic acid; RNA, Ribonucleic acid; TLR, Toll-like receptors; APC, Antigen-presenting cells; Ig, Immunoglobulin. (Picture was Created with BioRender.com).

The TLR9-related immune system plays a crucial role in developing vaccines against viral infectious diseases. TLR9 has a restricted cell expression profile, limited mainly to dendritic and B cells and, to some extent, in monocytes/macrophages, neutrophils, and T cells (80). Synthetic TLR9 agonists, CpGODNs, have been investigated as vaccine adjuvants for a long time. Synthetic TLR9 agonists, such as CpGODNs, have been extensively studied as vaccine adjuvants. Several vaccines containing CpGDNAs, such as Heplisav-B for hepatitis B and MVC-COV1901 and Corbevax for SARS-CoV-2, are already in clinical use. Recently, a combination of TLR9 and STING agonists as adjuvants has shown promising results, effectively increasing the immune response to intramuscular and intranasal administration of the SARS-CoV-2 Receptor-Binding Domain protein vaccine in preclinical studies (76). LC-Plasma, with its capacity to stimulate the innate immune system through the TLR9 receptor via CpGDNA-like components, similarly holds great potential as an immune booster (70) or as a novel prophylactic option for combating pathogenic viruses (**Figure 2B**).

### 4.4 Type-I IFNs therapeutic option

In a meta-analysis conducted by Buchynskyi et al., the administration of IFN-α during COVID-19 did not demonstrate consistent positive clinical outcomes, such as shortening the length of hospital stay or the viral elimination period (81). The lack of positive outcomes was attributed to differences in the dosing of IFN-α, variations in disease activity, and the heterogeneity of patients, including factors like genetics, age, and gender, which can impact the response to anti-COVID-19 treatments. Interestingly, it was observed that early intervention with IFN-α, within five days from the initial symptoms or at hospital admission, led to better clinical outcomes. On the other hand, late intervention with IFN-α was associated with a longer hospitalization period. This suggests that the timing of IFN-α administration plays a crucial role in determining its effectiveness in managing COVID-19 (81). It is important to note that while type-I IFNs are considered potential anti-viral agents, the response to IFNs in COVID-19 patients exhibited clinical variations based on the patients’ background characteristics. Improper timing and/or dosing of IFNs may exacerbate disease activity due to hyper-inflammation. Therefore, further research and a precise understanding of patient-specific factors are essential for optimizing the therapeutic use of IFNs in COVID-19 and other viral infection. Delayed type-I IFN responses, often triggered by factors such as viral persistence and prolonged inflammation, have been observed in elderly with high viral exposure (82). Such delayed responses can contribute to more severe disease outcomes in this age group. Moreover, individuals who have genetic mutations impacting the type-I IFNs pathways or possess neutralizing auto-antibodies against type-I IFNs might face challenges in effectively clearing the virus, thus, leading to persistent inflammation and more severe disease outcomes. On the other hand, young individuals with early and robust type-I IFN responses tend to clear the virus more efficiently and experience milder disease outcomes, especially in cases of low viral exposure (82). Endogenous IFN level is important in innate immunity. The baseline IFN secretion in the human body is associated with the commensal microbiota, and any disruption in this balanced ecosystem, known as dysbiosis, could lead to a decrease in the endogenous IFN level (6). Commensal bacteria provide stimulatory signals that keep immune cells constantly detecting and responding to potential viral threats. Unfortunately, while effectively targeting pathogenic bacteria, use of antibiotics can indiscriminately kill or significantly reduce the number of beneficial commensal bacteria responsible for sustaining the IFN signals and the related antiviral state (83). A noteworthy report highlighted a clinical correlation between prior antibiotic exposure and increase in severity of COVID-19 was observed in Spain (84), underscoring the potential impact of dysbiosis and the importance of maintaining a healthy commensal microbiota for a robust antiviral defence.

Therapeutic use of exogenous IFN-α administration needs to address the concern of potential adverse effects. IFN-α treatment is typically reserved for patients with severe viral infections, such as the Hepatitis C virus. While IFN-α can effectively treat these infections, it is also associated with various adverse events that require careful consideration. The most common side effects include influenza-like symptoms such as fever and fatigue, which are linked to non-specific activation of the inflammatory response. Other reported adverse effects include digestive symptoms and hematologic toxicity. Although most of these adverse events are of moderate intensity, they can still be unpleasant and painful for patients receiving associated with IFN-α treatment (85).

The prophylactic use of IFN-α as a preventive measure against SARS-CoV-2 infection is not considered rational due to the potential adverse events and the associated financial burden. However, consumption of LC-Plasma presents a promising alternative as it has shown the ability to activate pDCs safely without causing adverse events (**Figure 2A**). LC-Plasma is a probiotic that has been evaluated for safety in adults (86) at five times the normal volume and in small children aged 6 to 8 (35). This makes it a potentially safer option for immune modulation in the context of COVID-19 prevention and perhaps other viral infection.

### 4.5 The role of probiotics in Post-COVID-19 era

In summary, to effectively combat COVID-19 and other emerging viruses, it is crucial to understand the role of the innate immune system, specifically pDCs, and their ability to produce the antiviral cytokine IFN-α. Here two strategies are proposed: 1) boosting the innate immune system and 2) enhancing IFN-α production/utilization in the host. Probiotics play a significant role in these strategies, as they have the potential to improve the immune system and reduce the severity of viral infections, including COVID-19. The lung-gut axis is also important to consider, as intestinal microbiota conditions can influence the severity of COVID-19, and the use of oral antibiotics may worsen COVID-19 symptoms (87).

Recent development of our studying of COVID-19 has revealed a new syndrome named ‘Post COVID-19 condition (Long COVID),’ which is defined as ‘the illness that occurs in people who have a history of probable or confirmed SARS-CoV-2 infection, usually within three months from the onset of COVID-19, with symptoms and effects that last for at least two months’ (88). These symptoms include fatigue, cognitive disturbances, chest pain, dyspnea, arthralgia, and a decline in quality of life (89). Based on previous report, Long COVID may be influenced by the composition of their gut micribiota, and consequently, damaged gut microbiota or dysbiosis can act as modulators of systemic inflammatory activity and may impact various organs through the multiple gut-organ axis (90).

Probiotic, LC-Plasma shows promise in activating pDCs and inducing “endogenous” IFN-α at appropriate levels to prepare the body against viral infections. It is suitable for use in healthy individuals and vulnerable populations, such as the elderly and pediatric patients, as it does not cause adverse effects. LC-Plasma is already available as a food supplement in various countries, such as Japan, Vietnam, Singapore, Europe, and the US, LC-Plasma was already commercially available as a food supplement, functional food, or yogurt, making it a feasible, affordable, and safe option for countering viral infections worldwide. However, further in-depth and comprehensive research is necessary to fully understand the potential of LC-Plasma and other probiotics in the fight against viral infections.

## 5 Conclusion

This review examines the clinical effects of probiotics on viral infectious diseases from pre-COVID-19 to mid-COVID-19 and post-COVID-19. In the pre-COVID-19 era, probiotics were extensively studied for their impact on gastrointestinal infections, common colds, and the flu. While they showed clear efficacy in treating gastrointestinal infections, their effectiveness in healthy individuals was sometimes difficult to demonstrate. During this time, except for LC-Plasma, the mechanism of action of probiotics against viral infections, was not well understood, particularly regarding their activation of pDCs and induction of IFNs via the innate immune system. As the COVID-19 pandemic emerged during the Mid-COVID-19 era, research on probiotics’ therapeutic effects for COVID-19 began as an adjunctive treatment. Several small-scale clinical trials reported improvements in gastrointestinal symptoms related to COVID-19, but their direct efficacy against COVID-19 symptoms and outcomes, such as viral clearance and hospital stay duration, were not consistently confirmed. Research is now exploring probiotics’ effects on the Post-COVID syndrome that occurs after COVID-19 infection. In the Post-COVID era, our understanding of the relationship between COVID-19 and the local innate immune system, including pDCs, has deepened. This knowledge is being utilized in developing new vaccines, research on immune activation using adjuvants like CpGDNA via TLR9, and new IFN-based treatments. The importance of the innate immune system in viral infections, especially pDCs as the first line of defense, has gained significance. Probiotics, particularly those like LC-Plasma that can induce anti-infectious factors, are being recognized as potential adjunctive immunoregulatory agents to complement drug treatments for viral infections. As we continue to deepen our understanding of the immune system and the interaction between probiotics and the innate immune system, probiotics are expected to play an increasingly important role as auxiliary therapeutics against viral infectious diseases. Their ability to induce anti-infectious factors, like LC-Plasma, offers promising future therapeutic and prophylactic options for viral infections.

## Author contributions

OK: Conceptualization, Data curation, Funding acquisition, Supervision, Validation, Visualization, Writing – original draft, Writing – review & editing. ZL: Validation, Visualization, Writing – original draft, Writing – review & editing. KJ: Writing – review & editing. RT: Writing – review & editing. SA: Writing – review & editing.
